# Dietary practices and associated factors among pregnant women in West Gojjam Zone, Northwest Ethiopia

**DOI:** 10.1186/s12884-019-2702-z

**Published:** 2020-01-06

**Authors:** Yeshalem Mulugeta Demilew, Getu Degu Alene, Tefera Belachew

**Affiliations:** 1 School of Public Health, College of Medicine and Health Sciences, Bahir Dar University, P.O. Box 79, Bahir Dar, Ethiopia; 2 Department of Nutrition and Dietetics, Faculty of Public Health, Jimma University, P.O. Box 378, Jimma, Ethiopia

**Keywords:** Pregnant women, Dietary practice, Dietary diversity, Food variety score

## Abstract

**Background:**

The optimal dietary practice is a critical requisite for maternal nutrition. However, the majority of Ethiopian pregnant women have inadequate nutrient intakes. These may be due to their poor dietary habits. Identifying factors affecting the dietary practices of pregnant women is crucial to design appropriate interventions. In this country, the dietary practices of pregnant women and determinants are not well studied. Therefore, the purpose of this study was to assess the dietary practices and associated factors among pregnant women in West Gojjam Zone, Northwest Ethiopia.

**Methods:**

A community-based cross-sectional study was carried out among 712 pregnant women from May to August 2018. Quantitative data complemented with a qualitative method. Pregnant women were selected using a cluster sampling technique. Structured questionnaires were utilized for data collection. Data were entered into Epi-Info version 7.2.2 and exported to SPSS version 23 software for analysis. Data were described using frequencies and mean. A logistic regression analysis was done.

Three focus group discussions and 17 key-informant interviews were conducted for the qualitative data. Focus group discussion participants were mothers, husbands, and health professionals. Typical case and homogeneous sampling techniques were used for the key-informant interviews and focus group discussions, respectively. Thematic analysis was used for the qualitative data.

**Results:**

Only 19.9% of respondents had appropriate dietary practices. On the multivariable logistic regression analyses, being food secure [AOR = 2.25, 95% CI: (1.1, 4.5)], having high edible crop production [AOR = 2.00, 95% CI: (1.2, 3.2)] and a favorable attitude [AOR = 1.69, 95% CI: (1.1, 2.6)] were significantly associated with the appropriate dietary practices of pregnant women. In the qualitative study, lack of knowledge on maternal diet, cultural prohibition, and knowledge gap of the professionals were barriers that interfere with dietary practices during pregnancy.

**Conclusion:**

Pregnant women in the study area are found to have suboptimal dietary practices. Therefore, health professionals should give regular nutrition counseling using cards and role models for promoting diversified food production and consumption.

## Introduction

During pregnancy, women’s bodies undergo anatomical, physiological, and biochemical changes [1, 2]. These biological changes increase women’s nutrient requirements [2]. Thus, pregnant women should eat diversified foodstuffs that contain an adequate amount of energy, protein, vitamins, minerals, and water [3].

Despite this, the majority of pregnant women in developing countries have inadequate nutrient intakes compared to the standard recommended by the World Health Organization (WHO) [4, 5]. Especially, diets of Asian and African pregnant women are predominantly cereal-based with infrequent consumption of animal products, vegetables, and fruits [6].

Few studies done in Ethiopia have shown that nutrient intakes of pregnant women aren’t sufficient to meet their nutrient demand. Nutrient inadequacies are as a result of poor dietary practices (observable action of dietary habits) [7, 8]. According to the literature, good dietary practices of pregnant women vary within the country ranging from 26.9% in the Ambo district to 40.1% in the Gondar town [9–12].

To assess the dietary practices of pregnant women, some of the literature in the country used frequency of meal, while others utilized questions on the nutrient contents of the food which is not feasible to use in the Ethiopian context since the majority of pregnant women didn’t know the nutrient contents of the food. Nutrition information, education, monthly income, dietary knowledge, family size, age, ownership of radio, having an illness, and husband occupation were independent factors for the dietary practices of pregnant women [9, 10, 12, 13].

Inadequate dietary intakes are associated with intrauterine growth retardation, low birth weight, and premature delivery [14, 15]. These problems remained the foremost public health concern in Ethiopia and associated with the high neonatal and infant mortalities [16]. Conversely, excessive consumption of energy-dense foods linked with excessive gestational weight gain [17, 18] that, in turn, increases the risk of adverse pregnancy outcomes [19].

Taking this into consideration, the Ethiopian government continuously showed its commitment to avert nutritional problems by developing food and nutrition policy, strategy, and programs. Furthermore, the government designed the “Seqota Declaration” to end stunting by 2030 [20].

Despite the effort of the government to improve nutrition, few studies done on maternal nutrition in the country revealed suboptimal nutrient intakes of pregnant women compared with the standard recommended by the WHO [7, 8].

The dietary practices of pregnant women are influenced by a set of complex biological, physiological, psychological, economic, social, and environmental factors. Among these factors, individual (psychological and socio-demographic) and environmental (economic, social, cultural, availability, and accessibility of nutritious food) factors are amendable [21, 22]. Socio-demographic factors were assessed by previous researchers, whereas psychological and environmental factors affecting dietary practices of pregnant women were not assessed in the Ethiopian context.

Thus, this study adds to the literature on predictors of dietary practice by assessing environmental (enabling) and psychological (predisposing and reinforcing) factors. Enabling factors were livestock possession, edible crop, vegetable, and cash crop production. Predisposing factors were perceived susceptibility to and severity of the consequences of poor maternal diet, attitude, and intention to take a balanced diet, behavioral control, perceived benefit, and barrier of taking adequate diet during pregnancy. Reinforcing factors were family support to take a healthy diet, women’s autonomy, and subjective norms.

About 34.5% of pregnant women who attend antenatal care at public hospitals of Addis Ababa, Ethiopia reported good dietary practices. The same study also identified education, monthly income, attitude, and gravidity as factors associated with the dietary practices of pregnant women. This study used the above study as a reference to calculate the sample size using single and double population proportion formulas.

Identifying factors that affect the dietary practices of pregnant women is timely to design appropriate interventions. Therefore, the aim of this study was to assess the dietary practices and associated factors among pregnant women in West Gojjam Zone. The result will be a helpful baseline data about pregnancy & nutrition in the Amhara region, as it may be the first study of its kind in the region. It could be used as an input to policymakers and planners at the national, regional, and zonal level to design nutrition interventions important for improving maternal diet during pregnancy. The health service providers can take account of the findings of this study to modify the way nutrition education is delivered.

## Methods

### Study area and period

The study was conducted in West Gojjam Zone from May to August 2018. The zone is one of the 11 zones in the Amhara Region. In Ethiopia, region and zone are the first and second level administrative divisions, respectively. Zones are further divided into a number of woredas. Woredas (the third-level administrative divisions in the country) further divided into kebeles (the smallest administrative unit in the country).

West Gojjam Zone has 15 woredas with a total population of 2,641,240, of which 50.7% were females. The number of estimated pregnant women was 61,072. Teff, maize, millet, bean, pea, grass pea, pepper, barley, wheat, cabbage, collard green, tomato, potato, papaya, mango, and avocado are foodstuffs commonly produced in the zone. Cattle, chicken, sheep, and goat are the common livestock in the study area [23].

### Study design

A community-based cross-sectional study was done among pregnant women. In this study, quantitative data complemented with a qualitative method. A quantitative data collection followed by a qualitative method to explore and explain the result of the quantitative data.

### Source population and study population

All pregnant women in the zone were the source population, whereas pregnant women in the selected kebeles were the study population.

#### Inclusion criteria

Since dietary practice is affected by the local culture, pregnant women who lived in the kebele at least for 6 months were included in this study. Gestational age before 16 weeks of gestation was another inclusion criterion because this is a baseline data of a cluster-randomized controlled community trial, which was registered in Clinical Trials.gov with the identification number of NCT03627156. Title of the trial is “the effect of guided counseling in improving selected nutrition outcomes of pregnant women in West Gojjam Zone, Ethiopia: A cluster randomized controlled community trial.”

#### Exclusion criteria

Pregnant women who had confirmed or diagnosed hypertension and/or diabetes mellitus were excluded from the study because nutrient requirements and dietary practices of hypertensive and diabetes cases are different from their counterparts.

### Sample size determination

The sample size was calculated using single and double population proportion formulas with their respective assumptions. The sample size calculated by a single population proportion formula using the following assumptions gave the largest sample size used in this study: 95% confidence level, 34.5% proportion of appropriate dietary practice based on previous study [13], 5% marginal error, 5% non-response rate, and design effect 2. The calculated sample size was 712.

### Sampling procedure

A cluster sampling technique was used to select pregnant women. From the 15 woredas in the zone, eight of them had a nutrition intervention program implemented by a nongovernmental organization. Therefore, these eight woredas were excluded from the study because dietary practices of pregnant women in these woredas may not be representative of the dietary practices of all pregnant women.

From the seven woredas, three (Bahir Dar Zuria, South Achefer, and Burie Zuria) woredas were selected by a simple random sampling (SRS) method. Based on proportional to the size allocation, ten clusters from Bahir Dar Zuria woreda, six clusters from South Achefer woreda, and another six clusters from Burie Zuria woreda were also selected using the SRS technique.

House to house survey was conducted to identify eligible pregnant women in selected clusters. All eligible women were included in the study. The women’s pregnancy status was assessed by inquiring about her last menstrual period and confirming with a pregnancy test.

### Data collection and measurements

Data were collected by interviewer-administered questionnaires. The questionnaire includes socio-demographic variables, obstetrics history, dietary related variables, food security status, maternal perception, and socio-cultural issues. Six female nurses and three male public health professionals were recruited as data collectors and supervisors, respectively. Additionally, three female laboratory technicians were recruited to do pregnancy tests. Data collectors administered the questionnaire through a face-to-face interviews at the participants' homes. To maintain the optimal privacy of the mothers, other family members didn’t have free access to the place where the interviews were conducted.

Food frequency questionnaire (FFQ), which was taken from previously validated questionnaires [24–26], containing 54 food items was used to collect dietary data. The questionnaire was also validated after the assessment of locally available foodstuffs. For the food frequency questions, the women were asked about the frequency of consumption of each food per day, per week or per month in the prior 3 months by taking the variation of dietary intakes within days of the week into consideration [25, 26].

Food items of the FFQs were grouped into nine food groups: 1. cereals, roots, and tubers; 2. Vitamin-A-rich fruits and vegetables; 3. other fruits; 4. other vegetables; 5. legumes and nuts; 6. meat, poultry, and fish; 7. fats and oils; 8.dairy; and 9. Eggs [24].The consumers of a food item were defined as the consumption of a food item at least once a week [26]. The number of food groups the women ate within a week were counted to analyze dietary diversity score (DDS).

Food variety score (FVS) was computed by counting the individual food items the women consumed within a week. Then, the mean FVS was analyzed. The utilization of animal source food (ASF) was assessed by counting the frequency of each animal source foods the women took within the days of a week. Finally, the frequency of ASF consumption was divided into terciles (three parts). Dietary diversity score, FVS, ASF consumption, and frequency of meal were used to assess dietary practices.

The food security status of the household was assessed using 27 questions, which were adapted from the household food insecurity access scale. The questions were previously validated for use in developing countries [27]. Food secure households experienced fewer than the first 2 food insecurity indicators. Whereas, a household which experienced from 2 to 10, 11–17, and > 17 food insecurity indicators were considered as mildly, moderately, and severely food insecure households, respectively.

The wealth index of the household was determined using Principal Component Analysis (PCA) by considering latrine, water source, household assets, livestock, and agricultural land ownership. The responses of all non-dummy variables were classified into three parts. The highest score was coded as 1. Whereas, the two lower values were given code 0. In PCA, those variables having a commonality value of greater than 0.5 were used to produce factor scores. Lastly, the score for each household on the first principal component was retained to create the wealth score. Quintiles of the wealth score were created to categorize households as poorest, poor, medium, rich, and richest.

To determine edible crop and vegetable production, each crop and vegetable species cultivated by the household were counted. The number of crops and vegetables produced was classified into three parts. The highest value was labeled as high production, while the two lower values were considered as low production. In the study area, khat, eucalyptus, “Gesho” (a local plant used to make tella or local beer and areki), pepper, and onion are the common cash crops. Count of these cash crops was used to decide cash crop production.

The total ownership of livestock was measured by Tropical Livestock Units (TLUs). There is no TLU index created specifically for Ethiopia, therefore, the indexes for Tropical Africa was used in this study. The TLUs were calculated using the following weighted index factors: cattle = 0.7, horses = 0.5, mules = 0.5, donkey = 0.5, sheep = 0.1, goats = 0.1, chickens = 0.01 [28].

Women’s autonomy was assessed using eight questions. For each question, code one was given when a decision was made by the woman alone or jointly with her husband, otherwise zero. The mean was used to classify a woman’s decision making power [16].

Maternal knowledge on diet during pregnancy was assessed using 12 questions. Code one was given for each question when the response was correct, or else zero. The attitude was assessed by 20 Likert scale questions using PCA. The factor scores were summed and ranked into terciles (three parts). Then the highest tercile was labeled as a favorable attitude, if not unfavorable attitude.

Subjective norms, intention, perceived susceptibility, perceived severity, perceived benefit, and perceived barriers were assessed using their respective composite questions. Mean was computed and women who scored above the mean for each variable were categorized as having subjective norms, intention, perceived susceptibility, perceived severity, perceived benefit, and perceived barriers, otherwise no.

### Quality assurance mechanisms

To maintain the quality of the research, the questionnaire was adapted from standard data collection instruments, and it was pretested. Data collectors, supervisors, and laboratory technicians were trained for 3 days. The data collection procedure was supervised by the supervisors and principal investigator.

The data collection team held a daily meeting, and feedback was given daily. Double entry and verification were done by the principal investigator. Important assumptions were checked using the standard procedures. The dietary practice was the dependent variable for the quantitative data. Socio-demographic, enabling, predisposing, reinforcing, and obstetric factors were independent variables.

### Data processing and analysis

Quantitative data were edited and coded manually. Data were entered into Epi Info version 7.2.2 software and exported to SPSS version 23 software for cleaning and analysis. The frequency of meal, FVS, ASF consumption, DDS, and dietary practice were determined.

Since a cluster sampling technique was used to select the study participants, a generalized linear mixed model was fitted to include cluster-level variables. The intercept-only model estimates the intercept as 0.272 (the average appropriate dietary practice across all clusters was 0.272 but wasn’t statistically significant (*p* = 0.068)). The intra-cluster correlation coefficient was closer to zero (0.077). This showed that 92.3% of the dietary practice scores were explained by women level variables. The non-significant variability in dietary practices of pregnant women at the cluster level could be due to considering the design effect of 2 during sample size calculation, which in turn increased the calculated sample size. Therefore, bivariate and multivariable logistic regression analyses were used to assess predictors. Hosmer-Lemeshow Goodness-of-fit-test was done to check model fitness. Correlation between independent variables was checked using the Pearson Correlation Coefficient. *P*-value < 0.2 was used as a cut-off point to select variables for the final model. Backward elimination was used, and *P*-value < 0.05 was considered statistically significant.

### Definitions of terms

-  ➢ Food is any nutritious substance that people eat or drink
-  ➢ Appropriate dietary practice: when women had at least four meals daily, good FVS, high DDS, and high ASF consumption, whereas it was inappropriate when women had less than four meals daily or poor FVS or low DDS or low ASF consumption [25].
-  ➢ High DDS: tertiles were calculated from food groups, and the highest tertile was considered as a high DDS, whereas the rest two lower tertiles were taken as a low DDS [26].
-  ➢ Food variety score: women who had above the mean food variety were considered as having good FVS, otherwise having poor FVS [25].
-  ➢ Utilization of ASF: the highest tertile for ASF consumption was considered as the high frequency of ASF consumption, whereas the two lower tertiles were taken as the low frequency of ASF consumption [26].
-  ➢ Knowledge of the women about maternal diet: women who score > =75 and > 50% from knowledge questions were considered as having high and medium knowledge scores, respectively. Or else labeled as having low knowledge score [12].

### Qualitative data

The dietary practice is an observable action of dietary habit, which is the lived experience of an individual. Therefore, a phenomenological qualitative study was conducted using key-informant interviews and focus group discussions (FGDs). Three FGDs involving 6–12 participants in each group were conducted in Amharic (local) language. Twelve FGD participants were mothers. Eight FGD participants were husbands, and six were health professionals.

Seventeen key-informant interviews were carried out among pregnant women, health professionals, nutrition officers (nurses and pharmacist in their profession), community leaders and one-to-five network member leaders (one-to-five network is a lower level governmental structure consisting of five people. One leader monitor, mobilize and train five members about health, nutrition, and developmental related issues). All participants were Orthodox Christians and Amhara in Ethnicity. The respondents’ age ranged from 25 to 55 years. The educational status of the qualitative study participants ranged from having no formal education to a primary degree (Table 1).

**Table 1:** Socio-demographic characteristics of qualitative study participants in West Gojjam Zone, Ethiopia

Variable	Key-informant interviews (*n* = 17)	Focus-group-discussions(*n* = 26)
Age in years		
<=30	7	5
31–34	5	7
35–39	1	8
> = 40	4	6
Sex		
Male	8	14
Female	9	12
Occupation		
Farmer	5	20
Clinical Nurse	4	
Midwife	5	6
Pharmacist(officer)	1	
Doctor	2	
Educational status		
No formal education	6	15
Primary education	1	5
Diploma	7	6
Degree	3	
Marital status		
Married	4	6
Unmarried	13	20

A typical case and extreme sampling techniques were used to select the study participants for the key-informant interviews. FGD participants were chosen using a homogeneous sampling technique.

The qualitative data were collected using the semi-structured interview and FGD guides, prepared by reviewing existing literature and the local context. Moreover, the guides were revised based on identified gaps during interviewing or facilitating. During data collection, tape-recorder was used in addition to field notes. All interviews were conducted by the principal investigator at a convenient time in public meeting halls in the village. The interviews took from 30 to 45 min. The FGDs were also facilitated by the principal investigator and took from 45 min to 1 h. Notetaker who participated in qualitative data collection before this research was recruited to take notes during the interviews and FGDs.

Transcription was done on the same day of data collection. During the interpretation of findings, individuals’ detailed views of the meaning of events were considered. To assure trustworthiness, key-informant interviews were triangulated with FGDs. The quantitative data were triangulated with the qualitative method.

First, the researchers repeatedly listened to tape-recorded interviews and FGDs. Then, verbatim transcription was done in the Amharic language. Transcribed interviews and FGDs were translated from Amharic to English. Finally, data were entered into the QDA MINER LITE version 2.0.2 software for analysis. Codes were given for similar ideas using the software and cross-checked between researchers, used to establish analytical categories. Then, the meanings were organized into themes for thematic analysis. Finally, the result was presented in narratives in triangulation with the quantitative results and illustrations.

### Ethical consideration

Institutional Review Board of Bahir Dar University approved the study. Permission was obtained from West Gojjam Zone and each woreda administrators. Written consent (fingerprint for women who couldn’t read and write) was secured from the study participants. Confidentiality was maintained by excluding personal identifiers from the data collection form and keeping the data in a locked board.

## Results

### Socio-demographic and obstetric characteristics of pregnant women

Seven hundred twelve eligible pregnant women were invited to participate in the study. Complete data were obtained from 694 study participants, with a response rate of 97.5%. The mean (± SD) age of women was 28.14 (±5.97) years. All respondents were Amhara in ethnicity and rural dwellers. Almost all (99.4%) women were Orthodox Christian by religion. The wealth index showed that only 19.6% of the respondents were in the richest category. One in five (20.5%) women had first pregnancy, and the mean (± SD) parity of women was 2.49 (±1.96) (Table 2).

**Table 2:** Socio-demographic and obstetric characteristics of pregnant women in West Gojjam Zone

Variables	Frequency (*n* = 694)	Percentage (%)
Age (years)		
< 20	42	6.1
20–24	138	19.9
25–29	207	29.8
30–34	171	24.6
> =35	136	19.6
Mean (+SD)	28.14(+ 5.97)	
Religion		
Orthodox	690	99.4
Muslim	4	0.6
Educational status		
Cannot read and write	520	74.9
Can read and write	42	6.1
Primary education	84	12.1
Secondary education	48	6.9
Occupational status		
Housewife	355	51.2
Farmer	339	48.8
Marital status		
Married	687	99.0
Single/ divorced	7	1.0
Husband education (*n* = 687)		
Cannot read and write	344	50.1
Can read and write	176	25.6
Primary education	120	17.5
Secondary and above education	47	6.8
Husband occupation (*n* = 687)		
Farmer	686	99.9
Government employee	1	0.1
Wealth index		
Poorest	126	18.1
Poor	151	21.8
Medium	139	20.0
Rich	142	20.5
Richest	136	19.6
Family Size		
< 5	358	51.6
> =5	336	48.4
Gravidity		
1	142	20.5
2–3	199	28.7
4–5	218	31.4
> =6	135	19.4
Parity		
0	153	22.0
1–3	331	47.7
4–5	159	22.9
> =6	51	7.4

### Dietary practices of pregnant women

**Fig. 1:**
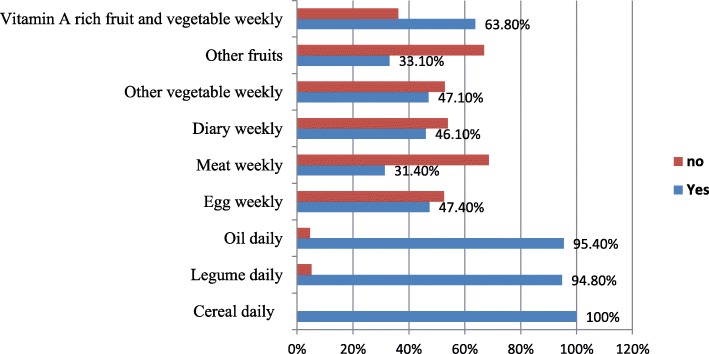
Consumption of foods by pregnant women based on food groups in West Gojjam Zone, Ethiopia

The dietary practices of pregnant women were shown in Table 3. The most frequently eaten foods were cereals, legumes, and oils but animal source foods, fruits, and vegetables were not often consumed by the study participants (Fig. 1).

**Table 3:** Dietary practice of pregnant women in West Gojjam Zone, Ethiopia

Variables	Frequency (*n* = 694)	Percentage (%)
Dietary practice		
Appropriate	138	19.9
Inappropriate	556	80.1
Women dietary diversity		
High	229	33.0
Low	465	67.0
Food variety score		
Good (above the mean)	322	46.4
Poor (mean & lower)	372	53.6
Utilization of animal source food		
High	254	36.6
Low	440	63.4
Frequency of meal/day		
> =4	245	35.3
< 4	449	64.7

### Factors associated with dietary practices of pregnant women

In the logistic regression analysis, Hosmer-Lemeshow Goodness-of-fit-test was 0.812. Pearson Correlation Coefficient was 0–0.2. Table 4 showed factors that had a statistically significant association with the dietary practices of pregnant women. Multivariable logistic regression analysis revealed food security, knowledge, attitude, edible crop, and vegetable production as the factors that were significantly associated with the dietary practices of pregnant women.

**Table 4:** Factors associated with dietary practices of pregnant women in West Gojjam Zone, Ethiopia

Variable	Dietary practice		COR (95% CI)	AOR (95% CI)
	Appropriate	inappropriate		
Get nutrition education				
Yes	56(8.1)	140(20.1)	2.02(1.3,2.9)	
No	82(11.8)	416(60.0)	1.00	
Food secure				
Yes	127(18.3)	417(60.1)	3.84(2.0,7.3)	2.25(1.1,4.5)
No	11(1.6)	139(20.0)	1.00	1.00
Intention				
Yes	95(13.7)	260(37.5)	2.51(1.7,3.7)	
No	43(6.2)	296(42.6)	1.00	
Knowledge on diet				
High	65(9.4)	108(15.6)	5.61(3.4,9.1)	3.23(1.9,5.5)
Medium	42(6.0)	159(22.9)	2.46(1.5,4.1)	1.81(1.1,3.1)
Low	31(4.5)	289(41.6)	1.00	1.00
Attitude				
Favorable	76(11.0)	154(22.2)	3.20(2.1,4.6)	1.69(1.1,2.6)
Unfavorable	62(8.9)	402(57.9)	1.00	1.00
Wealth index				
Richest	42(6.1)	94(13.5)	3.30(1.7,6.3)	
Rich	34(4.9)	108(15.6)	2.33(1.2,4.5)	
Medium	21(3.0)	118(17.0)	1.31(0.6,2.6)	
Poor	26(3.7)	125(18.0)	1.53(0.7,3.0)	
Poorest	15(2.2)	111(16.0)	1.00	
Family support				
Yes	66(9.5)	189(27.2)	1.78(1.2,2.5)	
No	72(10.4)	367(52.9)	1.00	
Edible crop production				
High	52(7.5)	91(13.1)	3.13 (2.0,4.7)	2.00(1.2,3.2)
Low	86(12.4)	465(67.0)	1.00	1.00
Household decision making				
Yes	105(15.1)	358(51.6)	1.76(1.1,2.7)	
No	33(4.8)	198(28.5)	1.00	
Perceived susceptibility				
Yes	92(13.3)	263(37.9)	2.22(1.5,3.2)	
No	46(6.6)	293(42.2)	1.00	
Perceived severity				
Yes	112(16.1)	374(53.9)	2.09(1.3,3.3)	
No	26(3.8)	182(26.2)	1.00	
Perceived benefit				
Yes	112(16.1)	345(49.7)	2.63(1.6,4.1)	
No	26(3.8)	211(30.4)	1.00	
Vegetable production				
High	72(10.4)	170(24.5)	2.47(1.7,3.6)	1.50(1.1,2.2)
Low	66(9.5)	386(55.6)	1.00	1.00

*AOR* Adjusted odds ratio, *COR* Crude odds ratio, *95%CI* 95 % confidence interval

In this study women who had medium level of knowledge were 1.81 times [AOR = 1.81, 95% CI: (1.1, 3.1)] and those who had high level of knowledge were 3.23 times [AOR = 3.23, 95% CI: (1.9, 5.5)] more likely to have appropriate dietary practices compared with those who had low level of knowledge. The likelihood of having appropriate dietary practice was 2.25 times higher among food secure women [AOR = 2.25, 95% CI: (1.1, 4.5)] than their counterparts.

Women who reside in the household with high edible crop production were 2.00 times [AOR = 2.00, 95% CI: (1.2, 3.2)] and those who had high vegetable production were 1.50 times [AOR = 1.50, 95% CI: (1.1, 2.2)] more likely to have appropriate dietary practices compared with their counterparts. Women with a favorable attitude had 1.69 times higher odds of having appropriate dietary practice than women with an unfavorable attitude [AOR = 1.69, 95% CI: (1.1, 2.6)]. In this study educational status, wealth index, livestock possession, and cash crop production had no association with the dietary practices of pregnant women (Table 4).

In the qualitative findings**, t**he three identified central themes were dietary practice, barriers of having appropriate dietary practice, and suggested solutions to improve maternal diet.

All respondents in the key-informant interviews and FGDs reported poor dietary practices of pregnant women. A female key-informant explained: *pregnant women frequently eat millet and maize injera (fermented pancake-like flatbread) with shero (stew). The amount and frequency of meals are less during pregnancy compared with their meals before pregnancy…*

A one-to-five-network leader, female key-informant also said: t*hough all foodstuffs are available at home, pregnant women eat maize injera with legume stew…*

### Barriers that hamper the dietary practices of pregnant women

The study participants mentioned a lack of knowledge, food insecurity, cultural prohibition, workload, unfavorable attitude, health professionals’ knowledge gap, and negligence as barriers affecting the dietary practices of pregnant women.

Thirteen key-informants and all FGD participants reported a lack of knowledge on diet during pregnancy as a predominant obstacle for having good dietary practice. A midwife key-informant reported: *in this area, pregnant women have a low level of knowledge on maternal diet. Due to this, they sell their nutritious foods and eat roasted grains throughout their pregnancy.*

The majority of the qualitative study participants stated that food insecurity was the main constraint of having appropriate dietary practice. A pregnant woman key-informant stated: *though this area is food secure, there are some women with poor living standards, for these women, food insecurity is the bottleneck to have appropriate dietary practices.*

Six key-informants and almost all FGD participants described the negative consequences of a cultural prohibition on maternal diet. There is no special diet culturally recommended for pregnant women in the study area rather the community suggested reducing the amount of meal per serving and avoiding some selected foodstuffs (linseed, pumpkin, and chickpea) during pregnancy. Moreover, taking an additional meal during pregnancy compared with the frequency of meals in her non-pregnant state was discouraged by the community. This cultural prohibition affects the dietary practices of pregnant women. The majority of FGD participants reported: t*he community suggests reducing consumption of food by considering that it predisposes to a big baby.* A pregnant woman key-informant said that *pregnant women avoid linseed, pumpkin, and chickpea due to cultural restriction...*

Another reason for having inappropriate dietary practice during pregnancy was the habit of practicing fasting in Orthodox Christians. Nearly all FGD participants reported: i*n this area, pregnant women abstain from eating animal products, and they do not take breakfast during fasting days. These inhibit them from taking an adequate diet.*

According to the qualitative study findings, a low level of crop production decrease availability and accessibility of foodstuffs in the household. These, in turn, determined women’s dietary practices. All FGD participants explained: *we can’t produce a variety of crops due to the shortage of land and the high cost of fertilizers. This restricts women from taking a variety of foodstuffs.*

Almost all respondents reported excessive workload and negative attitude of the women as constraints for having appropriate dietary practices during pregnancy. The majority of FGD participants claimed: m*ost pregnant women do farm activities in addition to their household duties, which hinder them from taking their meals on time. A s*ignificant number of FGD participants said: *pregnant women are not interested in preparing and eating their own diversified meals. They preferred eating a monotonous diet.*

All respondents reported a knowledge gap of professionals and negligence as the foremost obstacles to having appropriate dietary practice during pregnancy. A nutrition focal key-informant stated: *I am not doing anything to improve the maternal diet since I have no enough information about maternal nutrition. Not only I but also all other responsible bodies are not giving attention to maternal nutrition.*

A female, midwife key-informant said: *we give less time for nutrition counseling compared with other packages. We advise the women to take what is available at home by adding one extra meal because we don’t have enough knowledge about maternal nutrition.*

### Possible solutions to improve maternal diet during pregnancy

All respondents recommended the need for improving counseling methods. They proposed trimester based counseling. Qualitative study participants recommended using role models, counseling cards, and guidelines during counseling of pregnant women. Study participants also proposed developing nutrition guidelines with detail information about diet during pregnancy. Moreover, they suggested assigning nutritionists starting from the health center to a regional level. Furthermore, respondents proposed incorporating nutrition education in a school curriculum and giving refreshment training to health professionals.

A nutrition focal key-informant suggested: t*he method of nutrition education should be modified to improve the maternal diet. The counseling should be based on the trimester of pregnancy. It needs to be supported by role models, counseling cards, and guidelines. To do so, capacity building training should be given to health professionals. Besides, there is a need to assign a nutritionist with sufficient knowledge about nutrition at each level in the health system.* Another nutrition focal key-informant recommended as *the best solution to improve maternal nutrition is incorporating nutrition education in the school curriculum…*

## Discussion

Inappropriate dietary practice during pregnancy has been linked to the risk of adverse pregnancy outcomes. Despite this fact, in this study, only 19.9% [95% CI: (16.7, 22.6)] of respondents had appropriate dietary practices. Similarly, qualitative study participants reported poor dietary practices of pregnant women.

This result is much lower than the study findings in Northwestern [12] and Eastern Ethiopia [29]. The discrepancy might be due to differences in the study settings since the current study was conducted among rural residents with little access to nutrition education and health services. Moreover, respondents in this study had a low level of education. Their literacy level might have been a barrier to access information related to good nutrition practices.

Only 33.0% [95% CI: (29.5, 36.7)] of the study participants consumed a diversified meal. The minimum FVS was 46.4% [95% CI: (42.4, 50.3)]. Moreover, 36.6% [95% CI: (33.0, 40.0)] of respondents consumed ASFs. These findings are in line with previous study findings in developing countries [30]. According to the qualitative study findings, pregnant women frequently took a cereal-based monotonous diet. Moreover, they occasionally took fruits, vegetables, and animal products. These findings correlated with the study done in Northeast Ethiopia [31]. This indicates poor quality of maternal diets, which may significantly affect pregnancy outcomes [32].

Knowledge about diet during pregnancy plays a central role in determining women’s dietary practices [33, 34]. In this study, the likelihood of having appropriate dietary practice increased with increased maternal knowledge about diet during pregnancy. All respondents in the qualitative study claimed a lack of knowledge as a barrier that hampers the dietary practices of pregnant women. This finding is in agreement with the study findings in Bahir Dar Town [12] and Addis Ababa [13]. However, a study finding in Cameron reported a lack of association between knowledge and dietary practice, which is different from our study finding [35].

Production of edible crops and vegetables had an association with a better maternal diet. Limited production of crops and vegetables were barriers to having an appropriate maternal diet in the qualitative study findings. This finding is in line with the study finding in Southern Ethiopia [36]. The possible explanation might be that the crop and vegetable production affect the availability and accessibility of foodstuffs, which is the direct contributor for a good maternal diet [7, 22].

The likelihood of having appropriate dietary practices was higher among women who have a favorable attitude compared with their counterparts. This finding is supported by the qualitative study finding in which the negative attitude of the women was the reason for having a poor diet during pregnancy. This might be due to the fact that women who have a favorable attitude towards maternal diet are more likely to have the intention to take a balanced diet that directly affects their dietary practices [37, 38].

Food secured women had 2.25 times higher probability of eating adequate quantity and a high-quality diet compared with their counterparts. Similarly, in the qualitative study, the predominant reasons for poor maternal diet were unavailability and inaccessibility of foodstuffs. As per our study and previous study findings [39], food security is the primary prerequisite to improve maternal diet [40].

There was no association between livestock possession and dietary practices of pregnant women. The reason for this might be a high cost of animal products compared with plant foodstuffs. In this study, cash crop production was not a predictor for the dietary practices of pregnant women. This finding is consistent with the study finding in Ghana [41]. This might be due to the reason that cash crops are primarily produced for sale, not for home consumption.

This study makes important contributions to understand dietary practices and determinants of diet during pregnancy. It can be used as an input for policymakers, planners, researchers, programmers, and health professionals in the improvement of maternal diet to achieve the “Seqota Declaration” to end stunting by 2030.

The use of validated FFQ and supplementing the quantitative data with qualitative study findings were the strengths of this study. Although utmost efforts were made to reduce bias by giving intensive training for data collectors on how to probe the women to remember their dietary practices, the possibility of social desirability bias may be the limitations of this study. Key-informant interviews and FGD facilitations were held by the principal investigator with previous experience of qualitative data collection. The make-up of the focus groups may have influenced the discussion. But, we believe giving a clear introduction about the objective and probing during discussion promotes the active participation of all FGD participants.

## Conclusion

Pregnant women in the study area had suboptimal dietary practices. Having knowledge and favorable attitude, crop and vegetable production, residing in food secured households were predictors for having appropriate dietary practice. In the qualitative study, lack of knowledge, food insecurity, cultural prohibition, unfavorable attitude, professionals’ knowledge gap, negligence, and lack of a responsible body were reasons to have poor dietary practices.

### Recommendations

Nutrition education intervention needs to be tailored to meet the need for pregnant women to improve their dietary practices. Therefore, health professionals should give regular nutrition counseling using cards and role models. Nutritionists should be assigned at each level in the health system. Incorporating nutrition education into the school curriculum is recommended. Crop and vegetable production using available resources and backyard gardening should be encouraged by liaising with the agricultural sector. There is also a need to refresh the knowledge of health professionals on diet during pregnancy.

## Data Availability

The datasets analyzed during the current study are not publicly available because this study is a baseline study of a cluster-randomized controlled community trial that was registered in Clinical Trials.gov with the identification number of NCT03627156. The authors have using the baseline data for analysis of other objectives. But the datasets are available from the corresponding author on reasonable request.

## Abbreviations

ASF: Animal source food
DDS: Dietary diversity score
FFQ: Food frequency questionnaire
FGDs: Focus group discussions
FVS: Food variety score
GWG: Gestational weight gain
IUGR: Intrauterine growth retardation
LBW: Low birth weight
PCA: Principal Component Analysis
QDA: Qualitative Data Analysis
TLUs: Tropical Livestock Units
WHO: World Health Organization
